# Immunization enhances the natural antibody repertoire

**DOI:** 10.17179/excli2017-500

**Published:** 2017-07-10

**Authors:** Dylan Beinart, Daniel Ren, Cinthia Pi, Susan Poulton, Zoie E. Holzknecht, Chelsea Swanson, William Parker

**Affiliations:** 1 Duke University Medical Center, Box 2605, Department of Surgery, Durham, NC 27710

**Keywords:** immunology, cancer, vaccination, vaccine

## Abstract

The role of immunization in the production of antibodies directed against immunogens is widely appreciated in laboratory animals and in humans. However, the role of immunization in the development of “natural antibodies” has not been investigated. Natural antibodies are those antibodies present without known history of infection or immunization, and react to a wide range of targets, including “cryptic” self-antigens that are exposed upon cell death. In this study, the ability of immunization to elicit the production of natural antibodies in laboratory rats was evaluated. Laboratory rats were immunized with a series of injections using peanut extracts (a common allergen), a high molecular weight protein conjugated to hapten (FITC-KLH), and a carbohydrate conjugated to hapten (DNP-Ficall). Significantly greater binding of antibodies from immunized animals compared to controls was observed to numerous autologous organ extracts (brain, kidney, liver, lung, prostate, and spleen) for both IgM and IgG, although the effect was more pronounced for IgM. These studies suggest that immunization may have at least one unforeseen benefit, enhancing networks of natural antibodies that may be important in such processes as wound repair and tumor surveillance. Such enhancement of natural antibody function may be particularly important in Western society, where decreased exposure to the environment may be associated with a weakened natural antibody repertoire.

## Introduction

The term 'natural antibodies' describes immunoglobulin molecules produced against antigens without known history of immunization or infection (Schwartz-Albiez et al., 2009[26]). Natural antibodies are important for innate immune defense against potential pathogens and in the recognition and removal of abnormal cells (Grönwall et al., 2012[10]). It is the latter function that likely explains the significance of natural antibodies in tumor surveillance and cancer prevention (Umar, 2014[33]). Natural antibodies typically bind to the carbohydrate moieties and glycans expressed by precancerous and cancerous cells (Vollmers and Brandlein, 2009[36]), although natural antibodies also bind to a variety of “neoepitopes” that become exposed when autologous cells are disrupted (Ailus and Palosuo, 1995[3]; Casali and Schettino, 1996[5]; Guilbert et al., 1982[11]; Lacroix-Desmazes et al., 1998[12]; Logtenberg, 1990[15]; Lydyard et al., 1990[16]; Parker et al., 1997[21]; Quan et al., 1997[23]; Spalter et al., 1999[29]; Stahl et al., 2000[30]; Vassilev and Veleva, 1996[34]).

Current research suggests that the natural antibody repertoire is inherently linked to the host biome. For example, one of the differences between wild rodents and laboratory rodents is that the wild animals have much higher natural antibody levels compared to their laboratory counterparts (Devalapalli et al., 2006[8]). Recently Pi et al. (2015[22]) found that exposing laboratory rats to 'wild like' conditions partially reconstituted the natural antibody repertoire. This practice of exposing an organism to foreign antigens to manipulate immune function closely resembles the commonly used medical practice of vaccination, also known as immunization. Vaccination, one of the keystones of modern medicine, induces the formation of memory B-cells and antibodies that confer immunity to disease causing pathogens. Modern vaccination schedules expose patients to a variety of immunogenic compounds. Whilst primarily aimed at disease prevention, this exposure may have an unintended impact on the natural antibody repertoire.

This study examines the effect of vaccination on the natural IgM and IgG antibody repertoire. Sprague Dawley laboratory rats were given an immunization protocol designed to broadly stimulate the humoral immune system. The immunization cocktail included hapten conjugates with protein and carbohydrate carriers to provoke T-cell-dependent and -independent responses, respectively, as well as peanut extracts. The binding of natural antibodies from the sera of immunized and non-immunized rats to antigens extracted from rat organs was then quantitatively assessed.

## Methods

### Animals

Male (n=4) and female (n=8) Sprague Dawley rats were purchased from Harlan Sprague Dawley (Indianapolis, IN). The rats were acclimatized in standard animal housing at Duke University for 62 days. Once acclimatized, the rats were bred. The 31 female rats that resulted from the breeding were utilized for the experiments in this study. All animal housing and procedures were approved by the Duke University Medical Center Institutional Animal Care and Use committee.

### Experimental design

In order to evaluate the effect of immunization on the natural antibody repertoire, 20 of the 31 female rats were immunized with a cocktail containing peanut extract, fluorescein isothiocyanate labelled keyhole limpet hemocyanin (FITC-KLH) and 2,4-dinitrophenyl conjugated to AminoEthylCarboxyMethyl-FICOLL (DNP-Ficoll, Biosearch Technologies Inc. Novato, CA USA). The peanut extract was used to promote an IgE response, whilst FITC-KLH and DNP-Ficoll were used for T-cell-dependent and T-cell-independent stimulation, respectively. The remaining 11 rats acted as controls and did not receive any immunization. All rats were euthanized by CO_2_ inhalation at 71 days of age. Blood was collected from the posterior vena cava and centrifuged, and sera were stored at -80 °C until use.

### Production of peanut extract

The peanut extract was prepared as previously described by Pi et al. (2015[22]). Raw peanuts were lightly blanched at 100 °C for 3 min and pulverized using a BioPulverizer (Biospec Products, Inc. Bartlesville, OK, USA). Pulverized peanuts were then mixed with phosphate buffered saline (Roche, Indianapolis, IN, USA) at a 1 kg:4 L ratio. A homogenizer (OMNI GLH) was used to further homogenize the peanut-PBS mixture. The mixture was stirred overnight at 4 °C, then centrifuged for 30 minutes at 100 x g at room temperature. The aqueous fraction was centrifuged for 30 more minutes at 100 x g at room temperature to remove residual lipids. The remaining aqueous fraction, or “peanut extract”, was collected and stored at -80 °C until use. A Pierce BCA Protein Assay Kit (Thermo Scientific, Rockford, IL, USA) was used to determine the protein concentration. The extract was stored so that each aliquot was thawed only once prior to use.

### Synthesis of FITC-KLH

Conjugation and purification of FITC-KLH was conducted as previously described (Pi et al., 2015[22]). To begin with, 1.5 mL of 10mg/mL KLH (Sigma-Aldrich, St. Louis, MO, USA) was dialyzed against 2 L of 100 mM NaCO_3_ buffer at pH 9.3, in dialysis tubing (MWCO: 3,500; VWR Scientific, Rancho Dominguez, CA, USA). The mixture was kept at 4 °C for 24 hours and the dialysis solution was changed once during this process. FITC (Sigma-Aldrich, St. Louis, MO, USA) and KLH were then combined at a 20 µg:1 mg ratio, and incubated for 1.5 hours at room temperature. Next, the FITC-KLH conjugate was dialyzed in 500 mL saline solution overnight at 4 °C. The buffer was changed, and the sample was dialyzed for 2 hrs at room temperature. The absorbance of purified FITC-KLH was read at 280 nm and 495 nm, and the labeling ratio (FITC:Protein) was determined based on the ratio of those absorbancies. Two batches, with labeling ratios 2.4 and 5.3, were made and pooled. Labeled FITC-KLH was stored at -80 °C, in a manner that ensured each aliquot was only thawed once before use.

### Immunization cocktail

An immunization cocktail was mixed to provide broad antigenic stimulation with a single injection. Previously frozen aliquots of peanut extract, FITC-KLH and DNP-Ficoll were thawed and mixed in PBS, to concentrations of 2 mg/ml, 1 mg/ml and 2 mg/ml respectively. The resulting mixture was aliquoted and frozen to -80 °C until use.

### Immunization protocol

The immunization protocol consisted of 6 intraperitoneal injections over 14 days and is summarized in **Table 1**(Tab. 1). Rats were weighed and given the first injection at 43 days of age, designated day 0. This injection contained 1 mL/kg of the immunization cocktail and 1 mL/kg of Inject Alum (Pierce, Rockford, IL, USA) mixed for 30 minutes at room temperature on an Everlast rocker. Using the day 0 weight as the basis, the rats were given a 2 mg/kg body weight intraperitoneal injection of peanut extract on days 2, 4, 7 and 9. The immunization protocol concluded on day 14 with a final injection of 1 ml/kg of the immunization cocktail, without the Inject Alum. The resulting immunization schedule is detailed in Table 1(Tab. 1). All rats were euthanized 28 days after the first injection, at 71 days old.

### Preparation of tissue extracts

Tissue extracts were prepared by a method similar to that previously described (Pi et al., 2015[22]). These extracts provide autologous antigens for recognition by natural antibodies from the rat serum. For this purpose, 1 brain, 4 kidneys, 1 liver, 2 right lungs, 9 prostates and 7 spleens were collected from male WKY rats. The livers were washed with normal saline solution to remove excess blood. Perfadex solution (XVIVO Perfusion AB, Goteborg, Sweden) was used to perfuse the lungs via the pulmonary arteries. Other organs were not perfused. Once collected, individual organs were flash frozen with liquid nitrogen. All organs of the same type were pooled together and a Biopulverizer (Biospec products, Bartlesville, OK) was used to form a tissue powder. Tissue powders were then thawed to 4 °C and washed. The washing process began with the suspension of the tissue powder in 4.35 mL/g of PBS followed by mechanical agitation. This was followed by 1 minute in the centrifuge at 90 g, after which the liquid supernatant was removed. The brain, liver, lung and prostate powders were all washed twice. Due to the high blood content, the kidney powder was washed 4 times and the spleen powder was washed 5 times. After the final wash, PBS was used to adjust the concentration of each suspension to 0.18 gram of tissue powder/mL. The suspensions were then homogenized with two 45 second pulses in an OmnI GLH homogenizer (NW Kennesaw, GA). This process lysed cell membranes to expose sequestered intracellular antigens. The homogenized solutions were then spun at 9,170 g for 30 minutes at 4 °C. Finally, the liquid supernatant was collected, mixed with 10 % glycerol and stored at -80 °C. The entire process yielded 6 organ extracts with protein concentrations listed in Table 2(Tab. 2). Brain, kidney and liver extracts were diluted with PBS at 1:1.3, 1:2 and 1:3 ratios, respectively, prior to use.

### Preparation of PVDF membranes for immunoblot

Once collected, washed and homogenized, organ extracts were loaded onto PVDF membranes for immunoblotting. For this purpose, 280 µL of each organ extract (brain, kidney and liver, prepared and diluted as described above) was mixed with 108 µL SDS sample buffer (4x) (Novex NuPAGE®, Life Technologies), and 43 µL sample reducing agent (10x) (Novex NuPAGE®). Each mixture was vortexed, boiled for 7 minutes at 100 °C, vortexed again and centrifuged for 7 minutes at 15,996 x g. Subsequently, a 200 µL volume of each antigen mixture was loaded into the large well of each of two 4-12 % acrylamide gradient preparation gels (Novex NuPAGE® 4-12 % Bis-Tris ZOOM^TM^ Gel). The standard wells of each gel were loaded with 5 µL of a molecular weight standard (PageRuler Plus). Once loaded, protein electrophoresis with SDS PAGE was used to separate the antigen mixture. Brain and liver extracts were run at 45 V for 4 hr 50 min; kidney extract was run at 55 V for 4 hr; lung and prostate extracts were run at 65 V for 3 hr 15 min, and spleen extract was run at 70 V for 3 hr.

Once separated, antigens were transferred to PVDF membranes for 7 minutes at 20 V using an iBlot^TM^ gel transfer device (Ethrog Biotechnologies Ltd., Invitrogen). PVDF membranes were blocked for 70 minutes at room temperature using 1.0 bovine serum albumin, 0.1 % Tween 20, and 1x sodium azide in Tris buffered saline (blocking buffer). Each membrane was then cut into 16 strips (excluding the standard strip), each 4 mm wide.

### Immunoblotting

Sera were randomly selected from 8 of the 20 immunized rats and 8 of the 11 non-immunized rats. Membrane strips were incubated overnight at 4 °C with specific rat sera diluted 1/400 in blocking buffer. A control strip was incubated overnight in blocking buffer to account for anti-IgG and anti-IgM conjugate binding directly to organ-derived antigens. The next morning, the strips were washed 3 times for 10 minutes each with Tris buffered saline. Strips were then incubated for 1 hour at room temperature in alkaline-phosphatase conjugated, affinity purified goat antibody diluted at 1/1000 in blocking buffer. Goat antibody specific for the Fc region of rat IgG (Sigma-Aldrich) was used to detect natural IgG binding. Meanwhile, goat antibody specific for rat µ-chain (Sigma-Alrich) was used to detect natural IgM binding. After 1 hour, strips were washed another 3 times for 10 minutes each in Tris buffered saline. Once washed, 1-Step^TM^ NBT-BCIP (nitro blue tetrazolium and 5-bromo-4-chloro-3-indolyl-phosphate; Thermo Scientific) was used to develop the strips. Strips incubated in goat anti-rat IgG were developed for 28 minutes with fresh developer added after 14 minutes. Alternatively, strips incubated with goat anti-rat IgM were developed for 7 minutes. To conclude the process, all strips were washed with distilled water twice for 6 minutes each and air dried.

### Immunoblot analysis

Dried blots were scanned and Quantity One software v. 4.6.6 (Bio-Rad Laboratories) was utilized to quantify the substrate development on each image, corresponding to the amount of natural antibody binding to antigens. For each blot, the image intensity was manually adjusted to eliminate background noise, and the sensitivity was manually configured to maximize the congruency between the computer detected bands and the bands detectable to the investigator upon visual inspection. Similarly, the noise filter was adjusted to account for artifacts, typically small, that did not correspond to protein bands on the gel.

Natural antibody binding was quantified by several measures. The size of a single band was calculated by plotting a curve of the average intensity of all pixels in each row of the band against the band length. The area under this curve was taken as the size (Intensity x mm) of that band. Once all bands were identified, the size of the bands on the control strip was subtracted from the corresponding bands on all other strips. If the band size on the control strip was greater than the band size on an experimental strip, then this band was excluded from all analysis. The average band size associated with the serum of a given animal was defined as the average of all band sizes in a single strip, and the average band size for the group was calculated using the mean band size of all the blots in that group. The number of bands detected was taken as a measure of the number of antigens recognized by the natural antibody repertoire. Subsequently, the total natural antibody binding for a particular organ extract was taken as the sum intensity of all bands. Both total binding and band size were measured in arbitrary units of intensity that depend on developing time and exposure among other factors such as the nature of the enzyme conjugate. With that in mind, both measures were normalised to the mean of the non-immunized group. All calculations were performed for IgM and IgG antibody isotypes.

### Statistical analysis

The natural antibody binding characteristics of the sera from non-immunized and immunized rats were compared with unpaired, two tailed t-tests. All calculations were performed with Graphpad Prism Software (Graphpad Software, La Jolla CA).

## Results

### Average number of antigens recognized by natural antibodies from non-immunized and immunized rats

**Figure 1:**
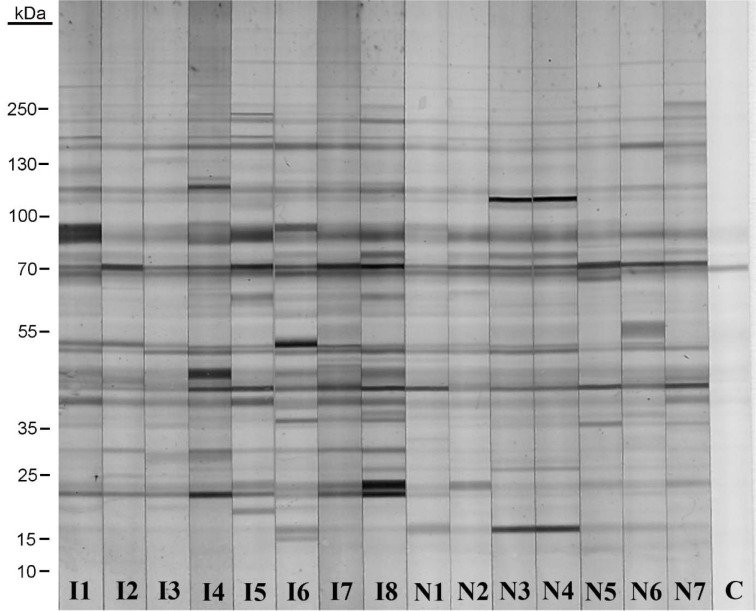
Binding of natural anti-rat brain IgM in the serum of immunized and non-immunized rats as evaluated by immunoblotting. Rat brain extracts were separated by SDS PAGE and probed by immunoblotting as described in the 'Methods' section. The analysis was limited to 15 animals (n = 8 immunized; lanes I1 through I8, and n = 7 non-immunized; lanes N1 through N7) due to size constraints of the gel. A control strip with no serum is labelled “C”, and indicates the reactivity of the anti-IgM conjugate with muscle-derived antigens.

**Figure 2:**
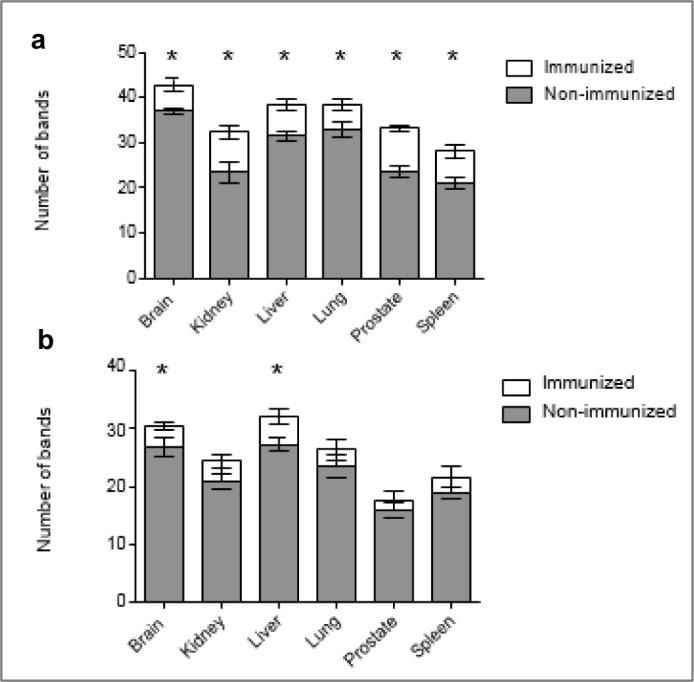
The natural IgM (top diagram) and IgG (bottom diagram) repertoire in immunized and non-immunized rats measured as the mean number of bands produced on immunoblot

A typical blot pattern using sera from immunized and unimmunized animals is shown in Figure 1(Fig. 1). The number of antigens recognized by natural antibodies was determined by immunoblotting and Bio-Rad Quantity One software, as described in the 'Methods' section. This number was taken as an indicator of the broadness of the natural antibody repertoire. This measure was evaluated for IgM and IgG from both immunized and non-immunized rats (Figure 2(Fig. 2)). The natural antibodies from the sera of immunized rats recognized a greater number of antigens from every organ extract, compared to the antibodies from non- immunized rats. The magnitudes of these differences were moderately isotype dependent. The differences in the number of antigens recognized by natural antibodies from immunized and non-immunized rats were larger with IgM antibodies compared to IgG.

Natural IgM from immunized rats, on average, recognized significantly more antigens than the natural IgM from non-immunized rats, regardless of which organ the antigens were from (Figure 2(Fig. 2)). Natural IgM from immunized rats recognized an average of 28 % more antigens per organ than did IgM from non-immunized rats. The greatest immunization-dependent difference in IgM recognition was seen with prostate antigens, where the IgM from immunized rats recognized an average of 40 % more antigens. In contrast, the smallest difference in IgM recognition between the two groups was seen with brain antigens, where the natural IgM from immunized rats recognized on average 16 % more brain antigens than natural IgM from non-immunized rats.

Natural IgG from the sera of immunized rats recognized an average of 14 % more antigens per organ than the natural IgG from the sera of non-immunized rats. Subsequently, the range of immunization-associated difference in IgG repertoire was also smaller than that of IgM. The IgG from immunized rats recognized 18 % more liver antigens than the IgG from non-immunized rats, and this was the largest difference observed. Meanwhile, the immunization-associated difference in the average number of lung antigens recognized by IgG was less than 1 % and not statistically significant. Furthermore, the immunization- associated differences in the number of antigens recognized by IgG were only statistically significant with respect to antigens from the brain and the liver.

### Average size of bands formed by natural antibody recognition of antigens in non-immunized and immunized rats

The average band size was quantified using Bio-Rad quantity one software as described in the 'Methods' section. It was then normalized to the average band size produced by the sera of non-immunized rats. This average band size was calculated for IgM and IgG binding in all organ extracts. Whilst band length is included, pixel intensity is the primary variable contributing to band size. The pixel intensity demonstrates how much antibody is bound to the antigen in that specific band. Therefore, average band size can be taken as the strength with which the antibodies bind to antigens. In this case, it represents the average strength of natural antibody binding to antigens extracted from particular organs.

**Figure 3:**
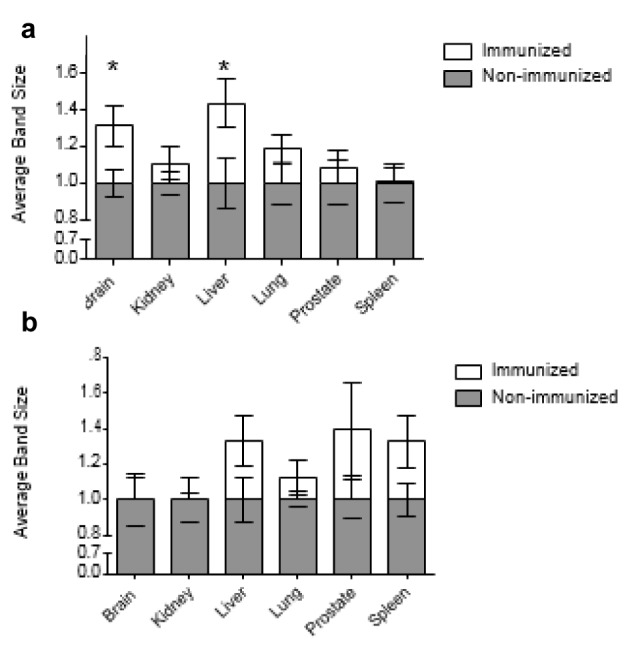
The average band size produced by the natural IgM (a) and IgG (b) from immunized and non-immunized rats binding to antigens from different organs

Taking the mean of the average band size seen with all organ extracts, the mean average band size produced by IgM recognition of antigens was 19 % greater with the sera of immunized rats compared to non-immunized rats (Figure 3(Fig. 3)). The average band size produced by IgM from immunized rats was equal to or greater than the average band size produced by IgM from non-immunized rats, regardless of the organ from which the antigens were extracted. The largest immunization-associated differences in average band size were seen with brain and liver antigens. With brain antigens, the average band size seen with IgM from immunized rats was 31 % greater than the average band size seen with IgM from non-immunized rats. Meanwhile with liver antigens, the immunization-associated difference in average band size was 44 %. No other organ extracts displayed statistically significant immunization-associated differences in average IgM band size. 

Meanwhile, the average band size produced by natural IgG binding in each organ extract was on average 18 % greater with the natural IgG from immunized rats as opposed to non-immunized rats (Figure 3(Fig. 3)). However, no organ extracts displayed a statistically significant difference between the average band size produced by the IgG from immunized and non-immunized rats. The maximum immunization-associated difference in average IgG band size was seen with prostate antigens. In this case the IgG from immunized rats produced bands that were on average 40 % larger than the bands produced by the IgG from non-immunized rats. Meanwhile when examining brain and kidney antigens, the average band sizes produced by natural IgM from immunized rats were marginally smaller than those produced by natural IgM from non-immunized rats (Figure 3(Fig. 3)).

### Total binding resulting from self-antigen recognition by natural antibodies from immunized and non-immunized rats

The total binding for each lane was calculated as the sum of all band sizes. The mean total binding for each group was assessed for IgM and IgG natural antibodies and normalized to the mean of the non-immunized group. The quantification of total binding reflects both the range of the natural antibody repertoire (number of bands) and the intensity of natural antibody binding (average band size). That is, the total binding can be approximated as by multiplying the number of bands by the average band size. Thus, an increase in either antibody repertoire or antibody binding strength can increase total binding.

**Figure 4:**
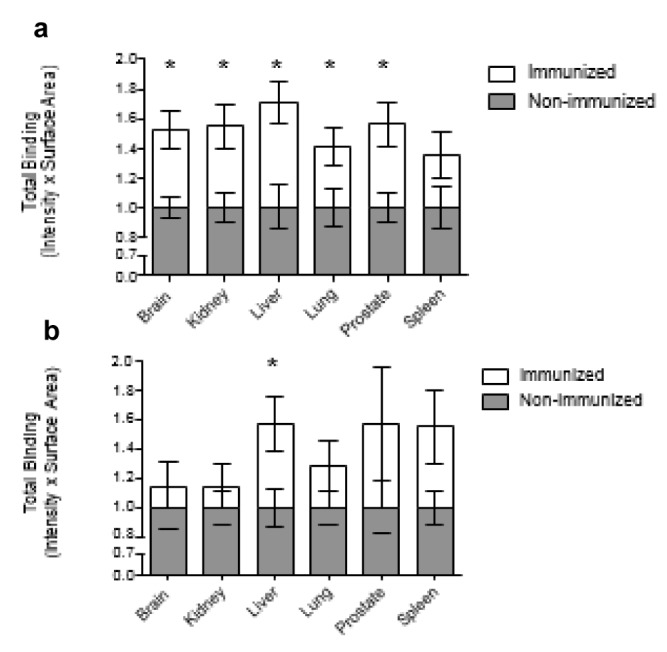
The mean total binding produced by the natural IgM (a) and IgG (b) from immunized and non-immunized rats binding to antigens from different organs

Compared to natural IgM from non-immunized rats, the total binding produced by natural IgM from immunized rats was significantly greater with all organ extracts except the spleen (Figure 4(Fig. 4)). The average of the immunization-associated differences in total IgM binding observed in all organ extracts was 52 %. In the liver, the natural IgM from immunized rats exhibited a mean total binding that was 71 % greater than that of IgM from non-immunized rats. 

It was noted above that total binding reflects the number of bands and the average band size. With antigens from the kidney, prostate and spleen, the relative immunization-associated changes in the mean number of bands recognized by natural IgM binding were larger than the relative differences in the average band size. Thus, the immunization- associated differences in total binding seen with self-antigens from these organs were more dependent on the expansion of the natural IgM repertoire as opposed to alterations in antibody binding intensity. In contrast, with liver and brain antigens it appears that the immunization-associated difference in total IgM binding was due in greater part to differences in the average band size and thus the intensity of antibody binding, although this finding was not statistically significant.

The immunization-associated differences in total binding of natural IgG to organ extracts were not as notable as the differences associated with IgM binding (Figure 4(Fig. 4)). The mean total binding of natural IgG to antigens from any organ extract was on average 38 % greater with IgG from immunized rats compared to IgG from non-immunized rats. With respect to antigens extracted from the liver, immunization was associated with an average of 57 % greater total IgG binding. This was the only statistically significant result observed with total IgG binding. Natural IgG recognition of prostatic and splenic antigens produced similar magnitude immunization-associated differences in total binding, but these were not statistically significant.

## Discussion

This study examined how a series of 6 immunizations over 14 days with a variety of antigens affected the natural IgM and IgG antibody repertoires of Sprague Dawley rats. On average, the natural antibodies from the sera of immunized rats produced a greater number of bands, larger average band sizes and greater total binding compared to natural antibodies from the sera of non-immunized rats. This indicates that in laboratory rats, immunization is associated with a larger natural antibody repertoire and stronger antibody binding, both of which contribute to greater total natural antibody binding.

Larger immunization-associated differences in natural antibody binding were seen with IgM antibodies compared to IgG antibodies. This fits with the current understanding of immunoglobulin class switching, where the acute phase of primary antigen exposure is characterized by a large IgM response. The short time scale of this experiment (28 days) may explain the isotype-dependent difference since the study was likely terminated before the completion of B-cell class switching. It might be expected that IgM levels would subside during the isotype switching process, coinciding with an increase IgG levels.

Whilst not as large, immunization-associated differences in IgG binding were observed. These changes may be a result of early isotype switching. Alternatively, IgG may have been expressed by B-cells previously primed to environmental exposure to antigens that cross react with the immunization antigens. The immunization-associated differences in IgG binding may also be due to T-cell-independent stimulation. DNP-Ficoll is the prototypical antigen for eliciting Type-2 thymus independent B-cell responses. *In vitro *and *in vivo *evidence has shown that DNP-Ficoll induces IgM and IgG responses.

The time scale of this experiment may also explain disparities in the magnitude of immunization-associated differences in the number of antigens recognized, compared to the differences in the average band size. Looking specifically at IgM, immunization was associated with a 28 % difference in the number of antigens recognized per organ extract, yet only a 19 % difference in the average band size per organ extract. Furthermore, the IgM from immunized rats recognized significantly more antigens in all organ extracts compared to IgM from non-immunized rats; whilst the immunization-associated difference in the average band size was only significant with antigens from 2 organ extracts. These observations suggest that in this experiment, immunization influenced the diversity of the natural antibody repertoire to a greater extent than it influenced increases in antibody binding to specific antigens. It is likely that the acute immune exposure used in this model led to polyclonal B-cell proliferation and an increase in the number of different antibodies produced. However, the acute nature of the exposure may not have allowed sufficient time for B-cell selection, somatic hyper mutation and the resulting affinity maturation that would have increased the strength of antibody binding. On the other hand, any affinity maturation that did occur may have been directed toward the antigens used for immunization without directly affecting the degree of binding toward unrelated targets. Future studies aimed at evaluating the effect of long term immunostimulation on the diversity of the natural IgG repertoire and on the binding strength of natural IgM and IgG antibodies may help further elucidate the processes involved.

The antigens in the immunization protocol were selected to ensure a robust and diverse humoral response. Peanut extract was used to provoke an IgE response whilst Imject Alum was added as an adjuvant to enhance the response. DNP-ficol and FITC-KLH were used to induce T-dependent and -independent B-cell responses. DNP-ficoll typifies Type-2 thymus-independent antigens in that it is a linear molecule with a repeating antigenic epitope (Mond et al., 1995[17][18]). This structure is similar to other T-independent antigens such as the capsular polysaccharides of the pathogenic bacteria *Neisseria meningitides*, *Streptococcus pneumoniae *and *Haemophilus influenza *(Mond et al., 1995[17][18])*. *Keyhole limpet haemocyanin (KLH) is a copper containing, oxygen transporting molecule derived from molluscs (Swaminathan et al., 2014[31]). It is a highly immunogenic T-dependent antigen used clinically for testing T-cell dependent immunity. KLH is commonly used for this purpose because adult populations have already been exposed to other readily available T-dependent antigens through immunization (Swaminathan et al., 2014[31]). These antigens include tetanus toxoid, hepatitis B surface antigen and inactivated influenza. Interestingly, tetanus toxoid is also used as a protein conjugate in juvenile *Haemophilus influenzae *vaccines.

The findings of this experiment have several implications for future research and potentially for clinical practice. The results indicate that it is possible to manipulate the natural antibody repertoire of laboratory rats by selective immunization. This can be accomplished relatively quickly (14 days) and without exposing the rats to any pathogens or live organisms. Whilst the immunization protocol did contain highly immunogenic compounds and an adjuvant, it nonetheless successfully influenced the natural antibody repertoire without the need for infection or exposure to potentially infectious organisms. Therefore, it may be possible to utilize immunization to manipulate the natural antibody repertoire in humans for the purpose of preventing or even treating disease.

As previously discussed, natural antibodies are important in innate immune defence as well as in cell surveillance and subsequently, cancer prevention. Indeed, non-specific effects of vaccination have been characterized in both animals and humans. These effects seem to have both positive and negative outcomes depending on such factors as gender and the specific nature of the vaccine schedule. Most studies have examined associations between immunization schedules and rates of illness across populations of children in low-income countries. For example, a randomized trial in children recruited through the Bandim Health Project suggested that, based on mortality rates by age 3, there may be a beneficial nonspecific effect of a two dose vaccination for measles at 4.5 months and 9 months, as opposed to the current approach of vaccinating against measles once at 9 months only (Aaby et al., 2010[2]). The DTP vaccination has an ambiguous history of oscillating between positive and negative associations, perhaps largely due to differences in methodology. Amongst the limited number of cohort studies that have been conducted, there have been mixed findings. Two landmark cohort studies found that DTP may diminish childhood mortality, but each assumed children were unvaccinated if no history was available, and one gave BCG and DPT together, potentially altering outcomes (Chan et al., 2007[6]; Fine et al., 2009[9]; Shann, 2010[27]; Vaugelade et al., 2004[35]). In contrast, a study in Guinea-Bissau compared vaccine schedule and gender differences among 626 pairs of male-female twins through mortality ratio measurements. Findings showed significant differences in mortality between those sets of twins that received BCG as their last vaccine versus DTP as their last vaccine, with mortality of the former nearly 29 times higher than the latter. It was also found that girls had a lower mortality rate, compared to boys, when measles or BCG was the last vaccine given, but a higher mortality rate when DTP was the last vaccine given (Aaby et al., 2004[1]; Shann, 2010[27]).

The above examples are only fraction of the research demonstrating heterologous effects of immunization on individuals from low-income countries (Benn et al., 2013[4]; Fine et al., 2009[9]; Shann, 2010[27]). However, there is a clear need for further research on the nonspecific effects of vaccination in higher income and post-industrial areas, which lack a majority of the diseases that are widespread in lower income countries. Among the limited vaccine research in higher income countries is a cohort study of Danish children, which found that receiving the live MMR vaccine as the last immunization on the vaccination schedule, compared with the inactive DTap-IPV-Hib, is associated with lower rates of hospital admissions for miscellaneous infections (Sorup et al., 2014[28]).

Despite the clear need for further research, conducting vaccine research on human subjects in higher income settings may face ethical and perhaps logistical hurdles. Alternatively, experiments using animal models offer a very feasible approach toward modelling the nonspecific effects of vaccines in humans living in high-income environments. Several animal studies have been conducted to examine immunology using wild caught and laboratory rats as models for humans living in pre-industrial countries and post-industrial countries, respectively (Devalapalli et al., 2006[8]; Lesher et al., 2006[13]; Trama et al., 2012[32]). Wild caught rats have significantly more natural antibodies than their laboratory counterparts (Trama et al., 2012[32]). Just as natural antibodies are produced to a greater extent in a natural or wild environment compared to a laboratory environment, it seems likely that humans in natural or pre-industrial conditions produce more natural antibodies than do Westernized humans.

It seems highly likely that Western society provides an environment that leads to an unhealthy understimulation of the immune system (Rook, 2007[25], 2009[24]). Thus it stands to reason that immune stimulation via immunization may provide beneficial nonspecific effects for high-income countries that it does not provide for low-income countries. An artificial stimulation of the natural antibody repertoire, for example, may be more important in Western culture than in a developing culture. Given that these natural antibodies are important for surveillance of diseases such as cancer, artificial stimulation of an underdeveloped repertoire that might exist in an environment having few natural pathogens seems warranted. 

In the future, the Western world will likely turn to artificial stimulation of immune function for health. Vaccination is only one form of such stimulation. Indeed, “helminthic therapy” is already being used by many (Cheng et al., 2015[7]; Liu et al., 2017[14]) in a Western world in which humans are largely devoid of helminths, and it is proposed that exposure to helminths may be necessary for normal immune function (Parker et al., 2012[20], 2013[19]). Testing the effects of artificial immune stimulation requires further research, particularly in order to elucidate how various forms of artificial immunostimulation might differentially influence immune function and health.

As Western society continues to make progress toward the eradication of infectious diseases, the possibility exists that immunization will transition from a tool to prevent infectious disease into a means of promoting healthy immune function and development. This study suggests that immunization may in fact be helpful in order to develop a natural antibody repertoire that is necessary for efficient surveillance of cancer, and that this function of immunization may be most critical in a hypothetical environment of the future which lacks infectious disease. 

## Figures and Tables

**Table 1:**
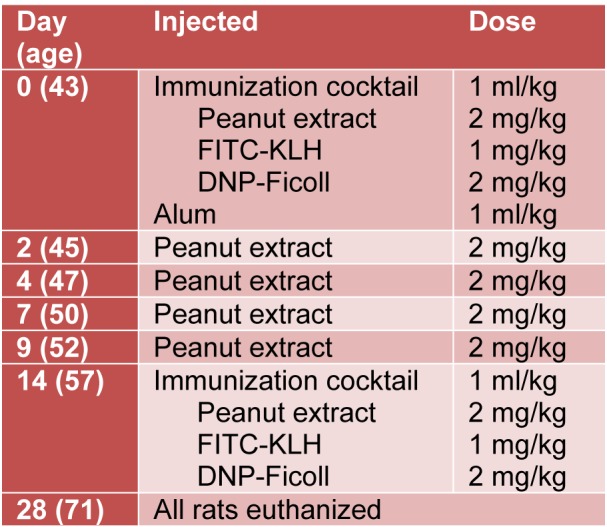
Dosing and schedule of immunization protocol

**Table 2:**
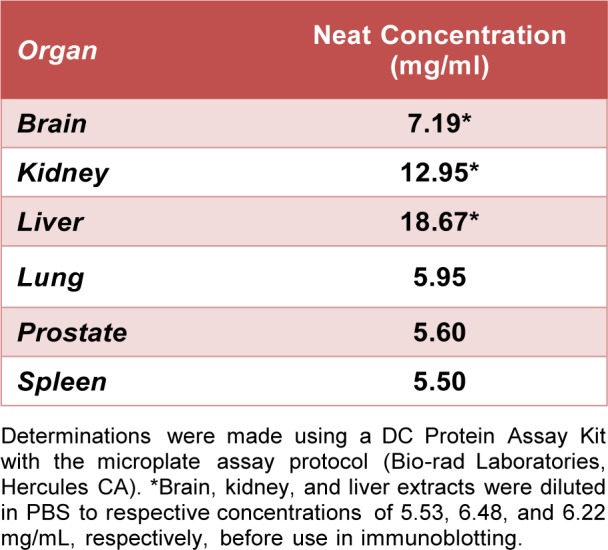
Total protein concentrations of organ extracts
